# Incorporation of explainable artificial intelligence in ensemble machine learning-driven pancreatic cancer diagnosis

**DOI:** 10.1038/s41598-025-98298-0

**Published:** 2025-04-23

**Authors:** Faisal Abdulaziz Almisned, Natacha Usanase, Dilber Uzun Ozsahin, Ilker Ozsahin

**Affiliations:** 1 Department of Information Systems, College of Computer and Information Sciences, King Saud University, Riyadh, Saudi Arabia; 2 Operational Research Centre in Healthcare, Near East University, TRNC Mersin 10, Nicosia, 99138 Turkey; 3 Department of Biomedical Engineering, Near East University, TRNC Mersin 10, Nicosia, 99138 Turkey; 4 Department of Medical Diagnostic Imaging, College of Health Science, University of Sharjah, Sharjah, UAE; 5 Research Institute for Medical and Health Sciences, University of Sharjah, Sharjah, UAE

**Keywords:** Biomarkers, Cancer, Diagnosis, Ensemble model, SHAP, Voting classifier, Cancer, Computational biology and bioinformatics, Biomarkers, Diseases, Health care, Oncology, Urology, Mathematics and computing

## Abstract

Despite the strides made in medical science, pancreatic cancer continues to be a threat, highlighting the urgent need for creative strategies to address this concern. Recently, a potential approach that has attracted significant attention is using machine learning in clinical decision-making. This research aims to analyze six machine learning algorithms, and an ensemble voting classifier, develop hybrid models for the early detection of pancreatic cancer based on several clinical characteristics and interpret their performance with Shapley Additive Explanations (SHAP). A publicly available dataset composed of 590 patient urine samples was utilized to develop six conventional models for the classification of cancerous from non-cancerous pancreatic cases through the analysis of specific attributes. An ensemble voting classifier was developed from the best-performed single models, which were later hybridized to form six novel hybrid models. The ensemble voting classifier outperformed all stand-alone models with an accuracy of 96.61% and a precision of 98.72%. The six novel hybrid models exhibited higher performance than single models with voting classifier random forest hybridized model outperforming others with an AUC of 99.05% (95% confidence interval (CI): 0.93-1.00) and an interpretation was given by SHAP showing top influential features in pancreatic cancer diagnosis that exhibited the greatest positive SHAP values. Employing rapid sophisticated models with high accuracy and precision holds significant promise in facilitating the effective detection of various diseases, including pancreatic cancer.

## Introduction

Over 400,000 men and women die from pancreatic cancer annually, representing a worldwide chronic disease^1^. The five-year survival rate for this disease is below 10% ^1^. Globally, this particular cancer ranks eighth among women and seventh among men in terms of cancer-related fatalities^2^. Consequently, by 2030, pancreatic cancer is predicted to replace lung cancer as the leading source of cancer death^1^. Before diagnosis, patients may have significant symptoms like pain, weakness, nausea, vomiting, and weight loss which may also indicate other health conditions^1^. As a result, it is a difficult oncology case because of its late-stage diagnosis and poor prognosis; however, early identification is critical for improving patient outcomes.

There is presently no viable, safe screening technology, whether molecular or imaging-based, that allows for detecting pancreatic cancer in asymptomatic individuals in its early stages. Traditional methods for identifying microscopic premalignant lesions; including endoscopic ultrasonography (EUS), positron emission tomography (PET), computed tomography (CT), and magnetic resonance imaging (MRI), are characterized by their high cost and restricted sensitivity and specificity^3^. Furthermore, the identification of small-localized lesions during a routine abdominal examination is challenging due to the deep anatomic position of the pancreas^4^. As a result, identifying pancreatic cancer biomarkers in physiological fluids (e.g., serum, plasma, urine, or saliva) is feasible at a reasonable cost, and requires minimal invasiveness, as a result, this enables earlier detection and diagnosis and consequently can facilitate prompt therapy planning^5^. Urine is a potentially useful substitute biological fluid to blood; which has historically been the primary source of biomarkers. Although lacking in dynamic range and proteome complexity compared to blood, it enables completely non-invasive sample collection^6^, high-volume analysis, and easy measurement repeats. Moreover, it is anticipated that at least some of the biomarkers would accumulate and eventually reach greater concentrations in urine as a result of ultrafiltration of blood by the kidneys. Biomarkers serve as prognostic, predictive, and diagnostic tools by reflecting physiological or pathophysiological processes associated with a disease. As a result, precise biomarkers can help with disease detection, early treatment planning, as well as drug discovery^7–9^.

Research by Fang et al.^10^ examined pembrolizumab-induced cholangitis as a side effect that occurs during cancer treatment with immune checkpoint inhibitors. They studied both clinical traits and treatment responses across patients receiving pembrolizumab therapy to find the specific risk factors that cause cholangitis development, and their findings indicate that early treatment-related problems require immediate diagnosis for cancer patients receiving immunotherapy. Similarly, Luo et al.^11^ conducted research on the clinical and therapeutic features as well as treatment responses of acute pancreatitis caused by pembrolizumab. Their study expanded knowledge about immune checkpoint inhibitor-related adverse outcomes, giving a reference for acute pancreatitis and treatment. They examined immunological markers that enable early prevention and management of adverse events in cancer patients getting immunotherapy-pembrolizumab by examining patients’ treatment responses and their case reports. Therefore, according to these studies, care should be taken in monitoring individualized treatment approaches thus increasing the emphasis to minimize cancer therapy-related complications by tracking biomarkers related to these adverse effects.

As computational models are recently being integrated into medical decision-making^12,13^, current technological advancements have opened up new possibilities for enhancing rapid detection and therapeutic decision-making. With an emphasis on their clinical implications and ability to completely transform medical care^14–16^, the current study examines the use of machine learning (ML); a subset of artificial intelligence (AI), algorithms in the classification of pancreatic cancer. ML models offer a unique opportunity to enhance the decision-making process in disease diagnosis and treatment^17^. In comparison to traditional diagnostic methods, ML techniques have the potential to detect pancreatic tumors at much earlier stages by analyzing and considering various clinical parameters, such as patient demographics, background history, serological and pathological tests, and other essential factors from which to draw diagnostic conclusions. Oncologists carry out intricate analysis of a substantial volume of data to diagnose pancreatic cancer using pathological slices and conventional imaging techniques, thus, the variations in educational background, training programs, as well as professional skills of physicians matter a lot when it comes to making diagnostic decisions.

Though ML algorithms demonstrate efficacy in generating accurate results and predictions, they encounter some limitations such as complexity and inadequate flexibility. These concerns may be very challenging in sophisticated medical conditions such as cancer, where minor errors can have a big impact on the final result. Clinical records often exhibit significant variability that leads to poor data quality. As a result, enhancing reliability and certainty in outcomes requires significant factors of clarity. The eXplainable Artificial Intelligence (XAI) aims to bring a fundamental change in the direction of AI, promoting more understanding of how model output is achieved and the relation between the data points in the dataset as well as their impact on the final result of the ML algorithms. Therefore, it is essential to establish an automated computer-aided system that is precise and requires minimal human interaction for pancreatic cancer diagnosis. With that in mind, this research aims to train and test six ML-based diagnostic classifiers that integrate numerous diagnostic parameters including biomarkers to classify pancreatic tumors and use best-performing classifiers in developing an ensemble voting model, the generated algorithm will be combined with each of the six single models to improve their performance in pancreatic cancer classification, furthermore, an XAI method; particularly Shapley Additive Explanations (SHAP), will be used in the interpretation of the model outcomes.

## Related literature

Pancreatic cancer remains among the most challenging cancers to diagnose early due to the lack of highly sensitive and advanced diagnostic techniques. Multiple researchers have applied AI approaches using molecular, biochemical as well as genetic features to increase pancreatic cancer diagnostic precision. Research conducted by Mahawan et al.^18^ built an ML framework that employed random forest, support vector machine (SVM), and eXtreme Gradient Boosting (XGBoost) to detect strong biomarkers for pancreatic ductal adenocarcinoma metastasis. The best-performing model had a 92.3% area under the curve (AUC). Similarly, Pu et al.^19^ performed pancreatic cancer diagnosis using XGBoost, light gradient boosting machine (LightGBM), and random forest which were trained and tested on plasma-derived extracellular vesicles-microRNA (miRNA) signatures, of which they achieved 95.8% accuracy. An approach comprised of multilinear principal component analysis and a quantum-simulated annealing algorithm (QSA) was proposed by Jiang et al.^20^ for classifying pancreatic illnesses. This methodology extracts eigentensors, employs brightness characteristics for high-order tensors, and applies SVM for classification, hence, the classification accuracy was 98.21%. Dang et al.^21^ applied the XGBoost, SVM, and random forest algorithms for the detection of the SLC6A14 biomarker which promotes pancreatic cancer metastasis through Wnt/β-catenin signaling. The findings showed that XGBoost had a high accuracy of 93.7%. Huang et al.^22^ performed a diagnosis of pancreatic cancer patients using circulating miRNA profiles through LightGBM and Random Forest techniques which resulted in an accuracy of 94.5%. The results from these researches highlight the potential of ML models and the benefits of biomarkers in pancreatic cancer diagnosis. However, the practicality of such studies in standard clinical practice might be restricted to facilities with minimal health equipment or regions of low income because the analysis was performed exclusively on data depending on invasive interventions.

Moreover, a weighted k-nearest neighbor (KNN) method was utilized by Kaya and Bilge^23^ to improve the classification of a dataset containing pancreatic tumors. This strategy used the t-test as a feature selection and feature weighting method. Consequently, as a result, the accuracy subsequently improved from 74.14 to 86.57%. Furthermore, ML applications in medical performance on a pancreatic malignancy database were thoroughly documented by Hayward et al.^24^, and methods for classification and prediction were both considered. The findings showed that data preparation, including feature selection and supervised feature discretization, might be utilized to greatly enhance the predictive effectiveness of various ML techniques as well as conventional multivariate regression techniques. In addition, the authors suggested that more performance metrics other than accuracy can be applied to assess the quality or robustness of the model.

Some other research studies focused on employing deep learning technology and merging different AI models for enhanced pancreatic cancer diagnostic tasks. Alaca^25^ developed a hybrid graphic MobileViT-based deep learning algorithm enhanced by Differentiable Architecture Search (DARTS), for image diagnosis of pancreatic tumors, achieving a 97.5% AUC-ROC score. Bakasa et al.^26^ developed a hybrid model combining deep neural network algorithms and conventional ML algorithms that yielded superior pancreatic cancer segmentations. Li et al.^27^ provide a computer-aided diagnosis system that uses PET/CT scans to detect pancreatic tumors, with an emphasis on segmentation, feature extraction, and classifier building. A hybrid approach, dual-threshold principal component analysis, and linear iterative clustering are some of the mechanisms used in the classifier. The model was tested on 80 PET/CT cases and had a 96.47% accuracy, 95.23% sensitivity, and 97.51% specificity in diagnosing pancreatic cancer. Furthermore, a public dataset was used to assess the pancreatic segmentation approach, and it exhibited superior performance compared to the alternative methods, as evidenced by its mean dice coefficient of 78.9% and Jaccard index of 65.4%. The effectiveness and precision of the computer-aided diagnosis model were verified using 10-fold cross-validation trials, outperforming previous techniques and showcasing its potential as a useful tool for early pancreatic cancer detection and therapy. Consequently, even though the hybrid AI models performed well, the detection of pancreatic cancer through plasma biomarkers and imaging tests remains challenging due to invasive processes involved in collecting data which makes their analysis more complicated than urine biomarkers.

A small number of studies employ ML models for assessing non-invasive urinary markers yet they rarely utilize XAI explainability approaches. Mikdadi et al.^28^ explore the use of AI in discovering biomarkers for rare cancer types, highlighting regulatory and ethical concerns. However, challenges like data quality, bias, and explainability in AI models are highlighted as a major gap in this field. The authors suggest the development of transparent and reliable AI models to advance research in these rare cancer types. The study by Acer et al.^29^ developed different urine biomarker-based ML models; Random Forest, SVM, and XGBoost which delivered the highest AUC-ROC score at 94.3%. They achieved high classification performance yet not all models reached their optimal level, highlighting the necessity of hybridized algorithms that potentially enhance robustness and generalization ability. Research conducted by Severeyn et al.^30^ detected early pancreatic cancer through urine biomarkers when employing naïve bayes, decision trees, and XGBoost. The XGBoost achieved the highest AUC-ROC results with a score of 96.2%. Similarly, using four ML approaches which included logistic regression, decision tree, random forest, and also gradient boosting, Ghosh et al.^31^ evaluated these ML techniques in pancreatic cancer diagnosis using urine biomarkers. The GBM model proved most effective for pancreatic cancer diagnosis through its AUC-ROC result of 97.2% demonstrating boosting approaches’ practical application in medical diagnostics. In addition, Kumar and Gayathri^32^ found random forest superior to XGBoost in pancreatic cancer detection through urine biomarkers achieving 93.8% accuracy. Though these studies were successful in ML model performance, they are limited in terms of ML interpretability for ease of understanding the ML results and their relation to the applied variables. These experiments are restricted in their clinical application potential since they lack the XAI methodologies required for transparent model decision-making in real-world clinical practice.

As observed in these few reported studies, there has been a considerable research breakthrough in oncology; specifically in the pancreatic cancer field, with the application of various AI methods however, there is still a gap in understanding the probable detection of pancreatic cancer with high accuracy and precision using urine biomarkers as well as the explainability on the performance of AI models on a beginner user level that even unexperienced personnel can easily understand the mechanism behind the model outcomes.

## Methods

### System methodology flow

**Fig. 1:**
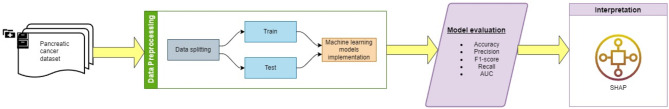
Experimental workflow.

To carry out the implementation of this project, a suitable dataset was selected for the development of the classification models. Once the information has been collected, the subsequent task involves preparing the dataset for better data quality and facilitating smooth processing by the models; mostly referred to as data preprocessing. This process involves handling missing values and data imbalance, as well as conducting label encoding, all of which pertain to this unique dataset. Thereafter, comes the second phase which is model building, which requires both the preprocessed dataset and ML methods. The algorithms used for classification include SVM, random forest, KNN, decision tree, naïve bayes, voting classifier, and logistic regression. GridSearchCV was applied for the hyperparameter tuning of the models to ensure their optimal performance. After developing six standard models and one ensemble algorithm, they were evaluated using five classification metrics; accuracy, precision, recall, AUC-ROC, and F1 score (see Fig. 1).

### Dataset description

The dataset used for classifying pancreatic cancer is sourced from Kaggle (https://www.kaggle.com/datasets/johnjdavisiv/urinary-biomarkers-for-pancreatic-cancer) and was originally assembled by Debernardi and his colleagues^33^. This specific dataset contains 590 rows and 14 columns which are all urine samples. The dataset contains various attributes such as ‘id’, ‘patient cohort’, ‘sample origin’, ‘age’, ‘sex’, ‘diagnosis’, ‘stage’, ‘benign sample diagnosis’, ‘plasma CA19 9’, ‘creatinine’, ‘LYVE1’, ‘REG1B’, ‘TFF1’, and ‘REG1A’. The output column ‘diagnosis’ contains values of ‘1’, ‘2’ and ‘3’. A value of ‘1’ signifies healthy patients; people who did not have any pancreatic conditions, cancer, or history of kidney conditions at the time of collection (183 samples), while a value of ‘2’ signifies benign cases (208 samples of which 119 cases were of chronic pancreatitis, 54 cases of gallbladder disorders, 20 cases of cystic lesions of the pancreas, and 15 cases with stomach discomfort and gastrointestinal symptoms that indicated a pancreatic origin), whereas a value of ‘3’ indicates malignant pancreatic conditions (199 samples). Every sample was obtained before surgery or chemotherapeutic intervention and was matched for age and sex, whenever feasible. More on the patient demographics and sample details can be found in^33^.

### Applied machine learning models

#### Logistic regression

Logistic regression (LR) is a widely used nonlinear ML tool used in fields like geology, biology, economics, medicine, and healthcare^34^. Its core component, the sigmoid function, transforms linear regression into a logit function, measuring the significance of predictors and their association direction. This function can determine probabilities by converting real-valued numbers into a range between 0 and 1, resulting in logarithmic probabilities for each independent variable^35^.

#### K-nearest neighbors

KNN is a powerful tool in pattern recognition, predicting object class based on its k nearest neighbors^35^. The distance of a data point is determined by the majority class of its k closest neighbors in the training dataset. However, KNN requires accurate distance calculations and optimal K value determination. It can be used with various datasets but may have computational costs and unnecessary features for large datasets^35^.

#### Random forest

Random Forest (RF) consists of several autonomous decision trees that are individually trained on a random selection of data. The trees are created throughout the training process, and the results are received from each decision tree. The algorithm concludes by using a technique known as “voting” to get the final forecast. In this approach, each decision tree votes for a certain output class, with the available classes being ‘cancerous’ and ‘non-cancerous’. The random forest algorithm selects the class with the highest vote count as the ultimate forecast^36^.

#### Support vector machine

SVM models, based on the concept of a “margin” on either side of a hyperplane, have become a significant advancement in supervised ML^37^. By increasing the margin to its maximum, the upper constraint on predicted generalization error can be reduced, allowing for the greatest feasible distance between the hyperplane representing separation and instances located on either side of it^38^.

#### Naïve Bayes

The Bayesian network, an ML classifier, is commonly used to solve classification problems. The Naive Bayes (NB) classifier is a basic type of Bayesian Network that utilizes the Bayes theorem, in which attributes are assumed to be independent of each other, given the class label. In the context of single-label classification, the algorithm computes the posterior probability for every group and assigns the specific case to the group with the highest probability^39^. It is commonly employed in ML projects to anticipate the classification of multiple classes^40^.

#### Decision tree

A decision tree (Dt) is a commonly used classification model that is created by making a series of hierarchical decisions about attributes to split data into smaller groups^41^. A subset is considered a pure partition if all of its instances fall under the same category. Once a subset becomes a pure split, it cannot be further split. If there are subsets that include a combination of different classes, there are two options: discontinue dividing the subsets and accept their impurity, or continue dividing the remaining subtree repeatedly^41^. While Dt may perform well in some classification applications, it might encounter challenges when dealing with a substantial number of classes and a scarcity of training data. Furthermore, it may be used with ensemble techniques to enhance performance even further.

#### Voting classifier

This is an ensemble model that aggregates the predictions of numerous independent models to provide a final prediction. Two methods may be employed: hard voting, which selects the majority class, or soft voting (the technique applied in the current study), which utilizes the average anticipated probability. This technique exploits the advantages of many models, resulting in enhanced overall performance and flexibility in comparison to individual models.

### Explainable AI

Our study introduces a transparent AI framework that assesses the most effective clinical variables for depicting cancerous and non-cancerous cases and capturing probable common characteristics. We employed a widely used XAI methodology, SHAP, to provide localized and comprehensive explanations for the predictions made by the trained models. SHAP is used to interpret the output of AI models by offering an extensive structure for assessing the relevance of features in model predictions by evaluating the value of the considered features and producing detailed interpretations of the behavior of a model. It utilizes game theory^42^ principles to provide explanations for the elements that impact a certain choice. SHAP may be employed for both local and global applications. Global explanation serves to clarify the overall behavior of a model, while local explanation is confined to a specific prediction inside the model^43^. It is beneficial because of its model-agnostic nature; however, a drawback of this approach is its increased computational time.

### Performance evaluation metrics

*Accuracy* measures the percentage of correctly classified cases, providing a general measure of a model’s performance, but can be misleading in imbalanced datasets.

$$\:Accuracy=\:\frac{True\:Positive\:+\:True\:Negative}{True\:Positive\:+\:True\:Negative\:+\:False\:Positive\:+\:False\:Negative}$$

*Precision* measures the proportion of true pancreatic cancer cases correctly identified by a model, crucial in clinical applications where false positives can cause excessive concern and additional testing.

$$\:Precision=\:\frac{True\:Positive\:}{True\:Positive\:+\:False\:Positive\:}$$

*Recall* measures the proportion of true positive predictions out of all actual positive instances. It is essential to ensure that most pancreatic cancer cases are identified.

$$\:Recall=\:\frac{True\:Positive\:}{True\:Positive\:+\:False\:Negative\:}$$

*F1-Score* is a measure of precision and recall, providing a harmonic mean that balances both metrics.

$$\:F1\:Score=\:2*\frac{Precision\:*\:Sensitivity}{Precision\:+\:Sensitivity}$$

*AUC-ROC* measures a model’s ability to differentiate between pancreatic cancer cases and non-pancreatic cancer cases, with higher values indicating better performance, ranging from 0 to 1. It uses true positive rates (TPR) and false positive rates (FPR) to measure the probability of a randomly chosen positive instance ranking above a randomly chosen negative instance. High TPR/recall and FPR/fallout indicate the proportion of positive data points correctly considered as positive and negative data points mistakenly considered as positive, respectively. Combining these metrics creates an ROC curve, with the area under this curve, AUC-ROC, representing the probability of a randomly chosen positive case ranking above a randomly chosen negative case.

$$\:TPR=\:\frac{True\:Positive\:}{True\:Positive\:+\:False\:Negative\:}$$

$$\:FPR=\:\frac{False\:Positive\:}{False\:Positive\:+\:True\:Negative\:}$$

*Confusion matrix* A confusion matrix; also called an error matrix, is an approach that assesses how well classification algorithms function. It thoroughly analyzes their performance in all categories, including accurate and inaccurate predictions. In addition to calculating assessment measures like accuracy, precision, recall, and F1-score, it assists in identifying misclassification instances. Confusion matrix analysis aids in determining the model’s advantages and disadvantages, therefore enhancing performance.

### Application of the proposed methodology

**Fig. 2:**
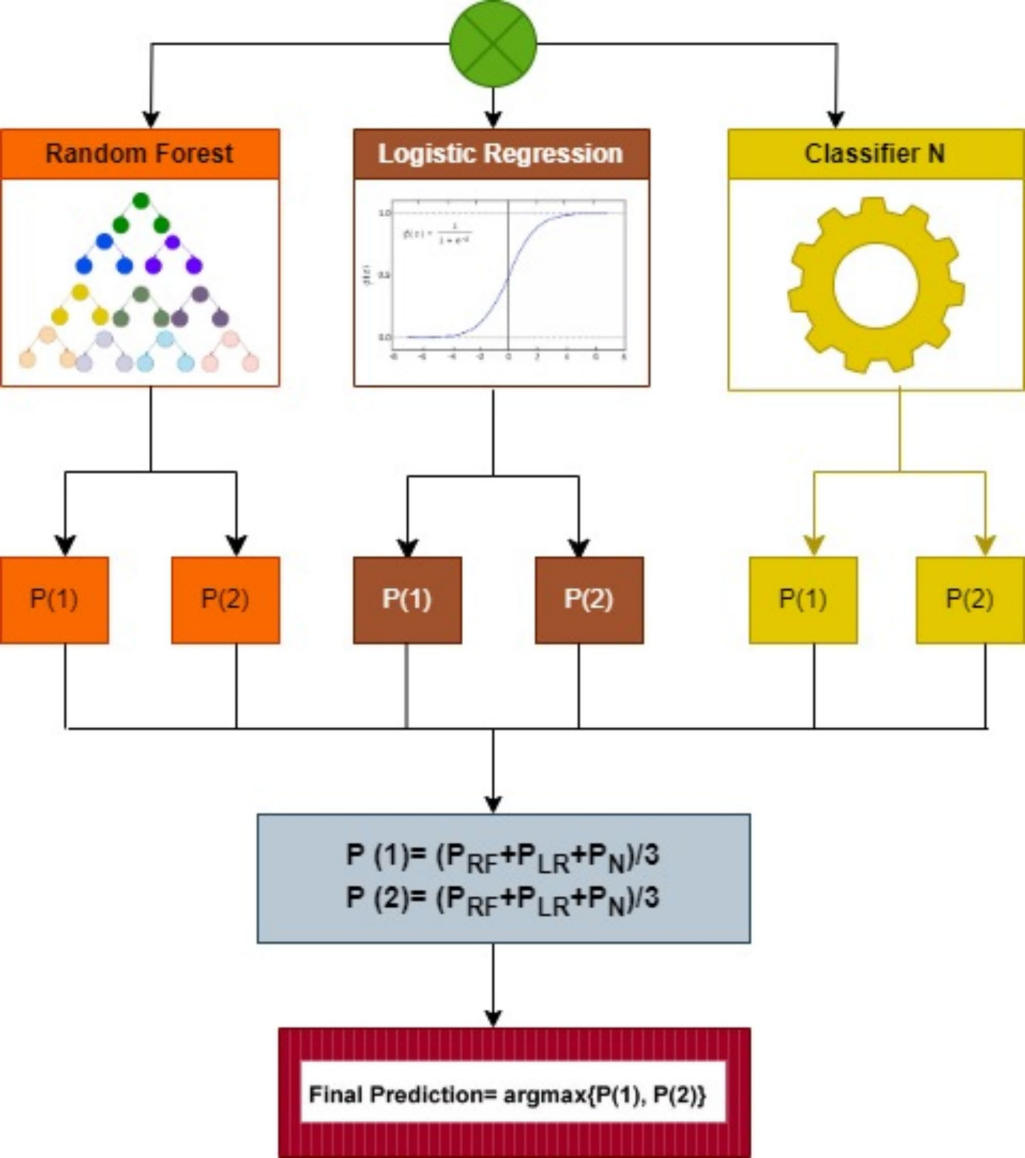
The architecture of the proposed voting classifier for pancreatic cancer diagnosis.

The initial stages involve data preparation for improved prediction quality and maintaining the integrity of data. We performed domain-specific filtering in addition to statistical encoding to optimize the biomarkers and clinical characteristic profiles in the analysis. The categorical variables were encoded with default labels while others were transformed into binary values (1: male, 0: female). This process is essential, especially in oncologic cases where parameters like tumor stages provide essential diagnostic insights. This encoding approach also allows algorithms to interpret categorical attributes correctly without creating inaccurate instances. Furthermore, numerical variables were handled using mean imputation techniques whereby missing information was replaced by the mean of the whole variable. Feature selection plays a vital role in boosting ML model efficiency and accuracy in oncological diagnosis that is often associated with incomplete and/ or unnecessary data or even duplicates within datasets. The model selection process focused on factors that are essential both clinically and physiologically, therefore variables that had no predictive importance such as “sample id”, “patient cohort”, and “sample origin”, were excluded. This filtering process helps prevent data loss which allows maximum benefit from the interpretability of the applied ML models while efficiently managing their complex structure. The six ML models were trained and tested on a public pancreatic dataset of urine biomarkers to classify pancreatic cancer. Commencing the training phase of all evaluated models, the dataset was divided randomly into 80:20, with the training phase using 80% of the data and the testing phase utilizing 20%, or 118 out of 590 samples, to classify pancreatic cases. When the selected features are fed into a classification model, the outcome will be either “cancerous” or “non-cancerous” (grouping healthy and benign conditions in one category). After obtaining each ML model’s evaluation metrics scores, the models are assessed to determine which models achieved an accuracy rate above 90%. A soft voting-based ensemble classifier (refer to Fig. 2) is developed and evaluated using highly accurate models with the same evaluation metrics described above. Thereafter, a hybrid model is built with the ensemble classifier and each one of the standalone conventional classifiers (voting classifier- (LR, DT, RF, SVM, NB, KNN)). The six ML models were chosen for hybridization considering different performance evaluation metrics, their computational cost, and their ease in interpretability as well as their diversity in their ability to classify different health condition cases using clinical features which is essential for real-world applications. RF and NB have proven over time to consistently deliver good performance in disease classification tasks thus both methods were chosen as appropriate benefiting models to be applied in the current study. Even though, SVM is associated with high processing costs, it was chosen due to its well-equipped systems to work with complex datasets. DT was chosen because of its straightforward decision process and its ease of interpretability factor. Though KNN can be computationally intensive, it was chosen due to its non-parametric nature and ability to handle complex datasets and detect data local patterns while LR is known for its simplicity which minimizes its computational cost and reliability in binary classification applications. A combination of such algorithms allows the development of a more robust classifier that incorporates the benefits from individual models while achieving optimized generalization, efficiency, and prediction which in turn increases complete diagnostic outcomes. The applied feature selection methods keep vital oncologic insights through successful high-performance computing and data dimensionality reduction, thus, real-life clinical environments benefit from this approach since it increases model generalization even when dealing with unpredictable data and missing values.

To demonstrate the rationale behind the final results of the models, we propose using SHAP which provides a visual representation of the specific contributions made by every attribute in the whole modeling process. According to the Shapley values concept^44^, a prediction or classification may be understood by considering each feature value as a “player” in a game, with the target serving as the reward^42^. Moreover, the implementation was carried out using a Jupyter Notebook version 6.4.5 on the Anaconda modules. The used workstation is a Windows 10 Pro that has an 11th -generation Intel (R) Core (TM) i7-11700KF, 3.60 GHz processor, 64.0 GB RAM, 64-bit operating system, 1 TB storage capacity, and video card GeForce RTX 3070.

## Results

The correlation between different attributes within a range of 0 to 1 was analyzed, whereby LYVE1 showed a high positive correlation with the output (“diagnosis”). The illustration also indicates that age, sex, plasma CA19-9, creatinine, LYVE1, REG1B, TFF1, and REG1A have a positive correlation in the diagnosis of pancreatic cancer meaning that they are statistically significant predictors of pancreatic cancer diagnosis. In contrast, stage, benign sample diagnosis features showed a negative correlation to the diagnosis. These parameters are used in ML-based systems to categorize pancreatic cancer cases.

Table 1 presents the outcome of four performance metrics, namely recall, precision, F1-score, AUC-ROC, and accuracy, in evaluating all trained conventional classifiers. The RF and NB models had an accuracy of 94.07% which was the highest among all the applied models, as a result, it is capable of properly diagnosing pancreatic cancer with higher accuracy than other algorithms. Conversely, the LR, KNN, SVM, and DT classifiers had lower accuracy ratings of 86.44%, 83.90%, 78.81%, and 89.83%, respectively, in comparison to the best outperforming models. RF continuously had a higher score in recall (equal to that of SVM), F1 score, and AUC-ROC metrics however NB outperformed RF in terms of precision as observed and bolded in Table 1.

**Table 1:** The results of the performance metrics of the six applied models.

Model	Accuracy	Precision	Recall	F1 Score	AUC-ROC
LR	86.44%	91.03%	88.75%	89.87%	89.54%
DT	89.83%	92.50%	92.50%	92.50%	88.36%
RF	**94.07%**	95.06%	**96.25%**	**95.65%**	**99.08%**
SVM	78.81%	77.78%	**96.25%**	86.03%	87.30%
NB	**94.07%**	**98.67%**	92.50%	95.48%	97.60%
KNN	83.90%	85.06%	92.50%	88.62%	90.30%

### Performance of the proposed ensemble models

**Fig. 3:**
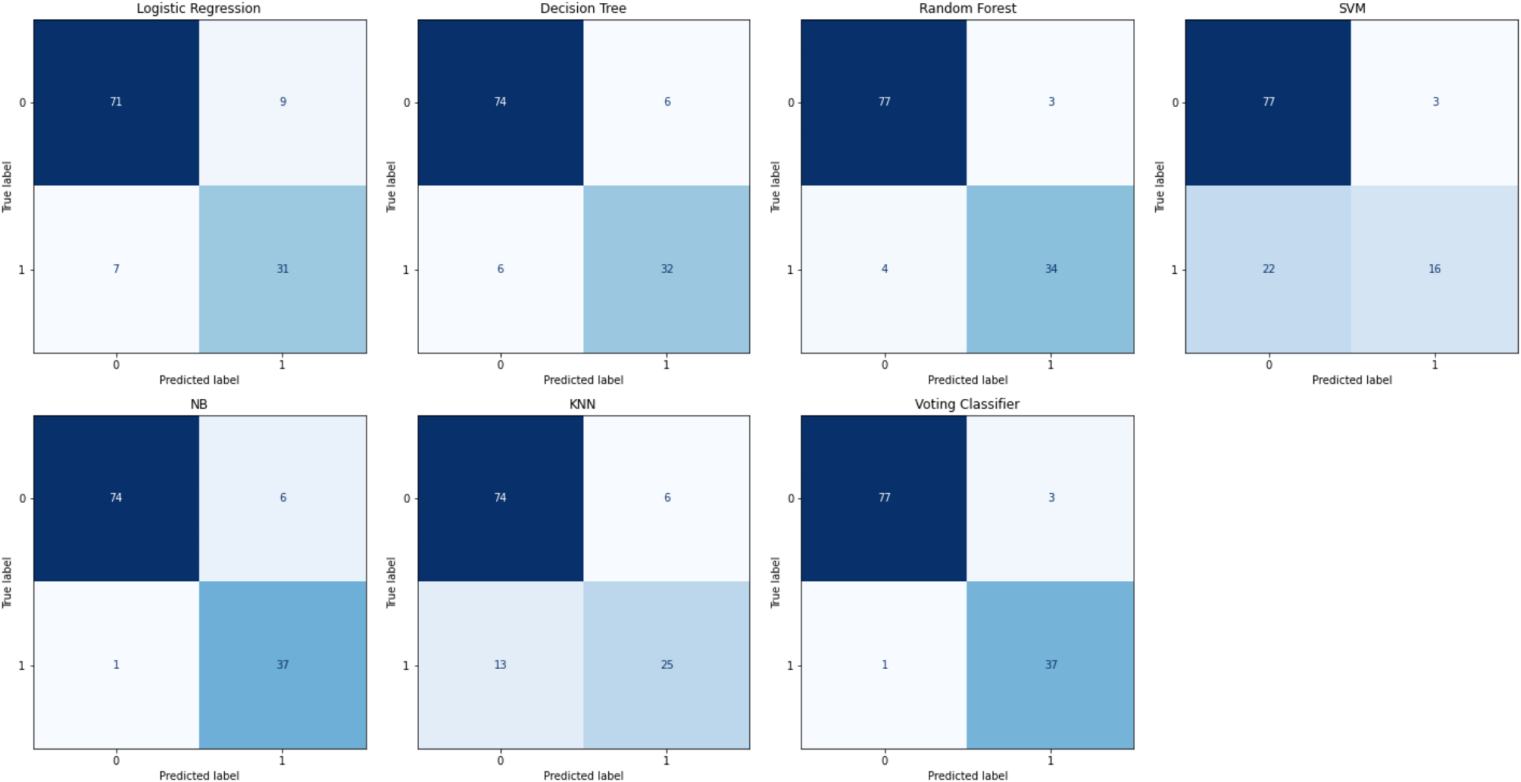
Confusion Matrix of all the six conventional models and the developed ensemble voting classifier.

Upon determining highly accurate models for pancreatic cancer detection, the next task involves designing an ensemble algorithm that incorporates the conclusions made by the conventional algorithms via the use of a voting policy; as previously mentioned in Sect. 3.6, because ensemble models enhance the performance of diagnostic frameworks, compared to single algorithms. As a result, the two most accurate single models (RF and NB) were used to build the ensemble paradigm. The ensemble classifier achieves an accuracy of 96.61% which surpasses that of all the six single models reported in Table 1. In addition to the improved accuracy, the values of other parameters are also superior to most of the ensembled models used in pancreatic cancer cases reported in the literature. The precision, recall, F1-score, and AUC-ROC values are 98.72%, 96.25%, 97.47%, and 98.98%, respectively (refer to Table 2). Unlike other applied standard ML models, the proposed ensemble model is highly specific and sensitive in detecting cancerous and non-cancerous cases as seen in the confusion matrix shown in Fig. 3.

**Table 2:** The performance results of the developed ensemble model in comparison to state-of-the-art ensemble models.

Model	Ensemble Model	Accuracy	Precision	Recall	F1 score	AUC-ROC
Ramachandra et al. ^45^	Rand index classifier with gradient descent	92.0%	-	-	-	-
Nené et al. ^46^	Stacking ensemble model	-	-	92%	-	91.0%
Reddy et al. ^47^	Boosting ensemble model	82.6%	-	91.0%	-	91.9%
Bakasa et al. ^48^	XGBOOST	94.0%	97.0%	96.0%	96.0%	95.0%
Yadav et al. ^49^	Random forest	92.5%	-	94.3%	-	93.0%
Lee et al. ^50^	Ensemble stacking classifier	77.0%	-	71.0%	-	74.0%
Li et al. ^51^	XGBOOST	69.0%		80.65%,		75.0%
**Our ensemble model**	**Voting classifier**	**96.61%**	**98.72%**	**96.25%**	**97.47%**	**98.98%**

**Fig. 4:**
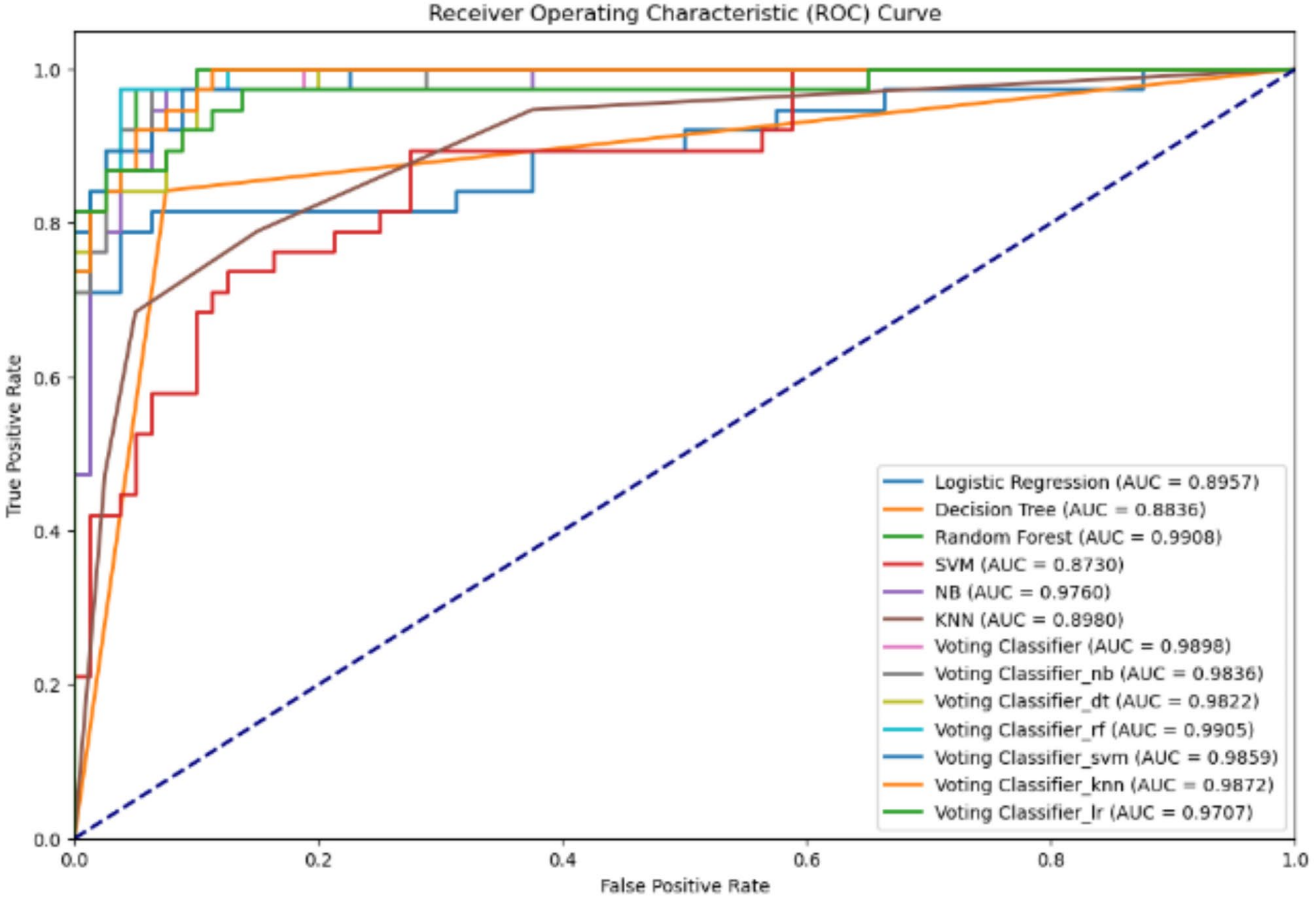
The AUC-ROC plot for both single algorithms alone and ensemble models.

The findings shown in Table 3 indicate that the hybridized novel paradigms exhibited a substantial increase in performance relative to the single models. While all six hybridized-novel paradigms effectively predicted and represented the sophisticated nature of the data more accurately than the single models, the Voting Classifier-NB, Voting Classifier-RF, and Voting Classifier-SVM models resulted in high accuracy in comparison with the other 3 hybrid models (Voting Classifier-DT, Voting Classifier-KNN, and Voting Classifier-LR). In addition, Fig. 4 illustrates the enhanced efficacy; in terms of the AUC-ROC curve, of the hybridized novel paradigms relative to the individual models in predicting pancreatic cancer cases. This underscores their effectiveness in precisely quantifying pancreatic cancer metrics demonstrating that the novel hybrid approaches have a higher positive impact on the performance of ML models in diagnosing pancreatic cancer than single algorithms.

**Table 3:** The prediction performance of the six developed hybrid models.

Hybrid Models	Accuracy	Precision	Recall	F1 Score	AUC
Voting Classifier-NB	**94.92%**	**98.68%**	93.75%	96.15%	98.36%
Voting Classifier-DT	89.83%	92.50%	92.50%	92.50%	98.22%
Voting Classifier-RF	**94.92%**	96.25%	96.25%	96.25%	**99.05%**
Voting Classifier-SVM	**94.92%**	95.12%	**97.50%**	**96.30%**	98.59%
Voting Classifier-KNN	94.07%	95.06%	96.25%	95.65%	98.52%
Voting Classifier-LR	93.22%	93.90%	96.25%	95.06%	97.11%

Additionally, Table 4 presents the performance of the hybrid ensemble voting classifier_RF used in this study, alongside results from prior literature, providing significant insights into the efficacy and dependability of ML algorithms for diagnosing pancreatic cancer using urine samples. LR in^52^ had a low accuracy score (76%) compared to all classifiers applied in this study. When compared to some deep learning models, such as convolutional neural network (CNN)^53^, the classification scores improved pancreatic cancer detection with higher performance metrics (95% accuracy) than our six single models but lower than the ensemble technique used in this study (voting classifier). Furthermore, our ensemble voting classifier outperformed all prior classifiers^33,52–54^ in terms of classification assessment criteria, with a maximum accuracy score of 96%.

**Table 4:** The comparative report of the current study with existing literature.

Study	Classifier	Recall	Precision	F1-score	Accuracy	AUC-ROC
Debernardi et al. ^33^	LR	0.83	-	-	-	-
Gerdtssona et al. ^55^	SVM	0.86	0.77			0.88
ALPU et al. ^52^	LR	0.64	0.89	0.74	0.76	-
Blyuss et al. ^54^	NN, RF, SVM, LR	0.81	0.90	-	-	0.94
Iwatate et al. ^56^	XGBOOST	-	-	-	-	0.68–0.80
Gress et al. ^57^	SVM	0.92	0.95		0.93	
This study	Ensemble Voting Classifier_RF	***0.96***	***0.96***	***0.96***	***0.95***	***0.99***

### Cross-Validation of the applied models

The efficiency of the proposed models was evaluated using K-fold cross-validation procedures. A model validation through cross-validation assesses both test results and model robustness. The method enables the evaluation of model performance across multiple data subsets. This work implements a 5-fold cross-validation technique because of its class balance features and better computational efficiency compared to other methods (i.e. 10-fold). This approach avoids performance bias when working with unbalanced data owing to its ability to maintain the original class distributions within each fold. In addition, when using a 10-fold method the performance estimates might become more consistent, yet this approach demands long training time while delivering limited accuracy improvement mainly for models with low variance such as LR and KNN. Therefore, the 5-fold method achieves optimal results in terms of computational expense and performance stability during both hyperparameter tuning and cross-classifier analysis procedures. The outcomes of the cross-validation in the current study reveal that the RF model continued to outperform other models, when evaluated individually and when combined with a voting classifier (Voting Classifier-RF hybrid model) with an average accuracy score of 0.92 ± 0.01 and an AUC of 0.98 (95% confidence interval [CI]: 0.87–0.99) according to cross-validation results shown in Table 5, proving to be statistically significant surpassing other models.

**Table 5:** Five-fold cross-validation results for stand-alone and hybridized models.

Single Models			Hybrid Models		
Model	Accuracy (Mean ± Standard deviation)	AUC	Model	Accuracy (Mean ± Standard deviation)	AUC
LR	0.84 ± 0.02	0.87	***Voting Classifier_lr***	0.89 ± 0.02	0.97
Decision Tree	0.87 ± 0.03	0.86	***Voting Classifier_dt***	0.87 ± 0.03	0.97
Random Forest	***0.92 ± 0.01***	***0.98***	***Voting Classifier_rf***	***0.92 ± 0.01***	***0.98***
SVM	0.77 ± 0.03	0.82	***Voting Classifier_svm***	0.89 ± 0.02	0.96
NB	0.89 ± 0.03	0.95	***Voting Classifier_nb***	0.91 ± 0.03	0.96
KNN	0.80 ± 0.02	0.83	***Voting Classifier_knn***	0.88 ± 0.03	0.97

### Interpretability

**Fig. 5:**
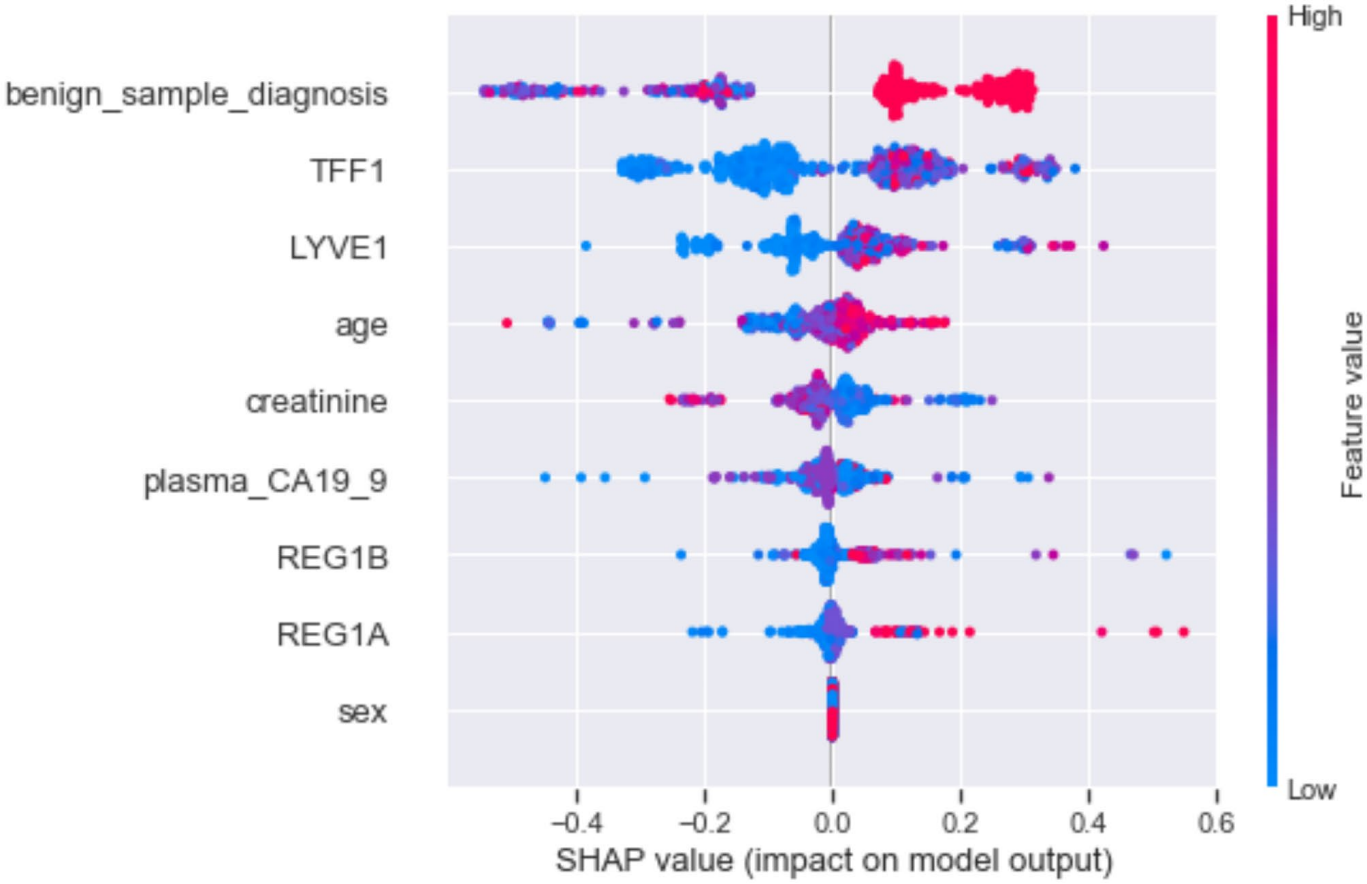
SHAP plot of all features from the applied model (SHAP global explanation and model summary).

Alternatively, our work used a widely-used XAI approach, SHAP, for understanding the classification process of the applied models. The SHAP summary plot in Fig. 5 displays the attributes arranged on the -axis based on their influence on the output of the model. This is determined by global SHAP values calculated for each data point in the dataset; plotted on the x-axis, which indicates the positive or negative impact of these features on the model performance (higher positive values indicate a greater likelihood of diagnosing early-stage pancreatic cancer, and lower negative values indicating an inferior probability). Furthermore, the attributes are arranged in descending order, with the most essential variables positioned at the top. Based on the plot in Fig. 5, it is evident that benign sample diagnosis, TFF1, and LYVE1 are the top three influential features of the target variable exhibiting the greatest positive SHAP values. This suggests that greater values of these three parameters have a substantial role in determining early-stage pancreatic cancer. In addition, the predictive model complexity directly impacts the computational requirement of SHAP analysis. Therefore, when applied to complex models such as ensemble and hybrid algorithms, SHAP faces extreme computational difficulties as it has to calculate feature contributions across a large configuration set. As a result, it becomes a bit challenging for real-time deployment, particularly in severe health conditions like cancer where SHAP models deal with computationally heavy tasks in producing explanations for high-dimensional data. Two possible solutions to achieve quicker turnaround time for oncologic cases include applying the SHAP Kernel Explainer technique or performing feature selection before SHAP analysis. Overall, the use of SHAP facilitated a more effective and visually clear representation of the significance of each feature, aiding in the identification of the major impact of biomarkers in cancer diagnosis.

## Discussion

The continuous advancement of the medical industry benefits from the robust data management capabilities of AI. Through AI innovations, the healthcare sector gained new diagnostic and prognostic assessment techniques that increased the probability of having efficient disease treatment outcomes. Furthermore, significant breakthroughs in AI-enabled scientific researchers to create modern tools that help them in risk assessment, screening, and diagnosis, as well as providing prompt successfully personalized treatment^58–60^. However, pancreatic cancer remains a challenging disease to diagnose and treat, with limited advancements in detection and treatment methods owing to its aggressive nature. To improve patient outcomes, non-invasive testing methods like urinary biomarker analysis are crucial to enhance early detection of this malignancy, regardless of symptoms. Consequently, recent studies demonstrate that ensemble-based ML models using multiple clinical and biochemical factors lead to increased diagnostic capabilities for disease models^61–64^. As a result, in this study, six ML models (LR, DT, RF, SVM, NB, KNN) were used to classify cancerous from non-cancerous pancreatic cases using urine biomarkers and other clinical parameters of which two of the models (RF and NB) that resulted in high accuracy were used to build an ensemble voting classifier. The results showed that the developed ensembled algorithm performed better than the two best-performing standard models as well as other applied algorithms with a 98.98% AUC-ROC score (reported in Tables 1 and 2) demonstrating its improved capability to predict results. Furthermore, the performance of the applied conventional ML models increased when combined with the developed ensemble classifier, with the Voting Classifier-RF hybrid model achieving a maximum accuracy score of 95.01% with an AUC of 99.04% (95% confidence interval (CI): 0.94-1.00). This applied novel diagnostic-model hybridization mode leverages the advantages of one algorithm to compensate for the limitations of another model, promoting better-performing health computational systems.

Our study aligns with multiple published works that detected pancreatic cancer using standard and/or hybrid AI approaches, providing essential information about parameters that affect the overall diagnosis therefore producing enhanced clinical diagnostic and therapeutic insights. Baba et al.^65^ analyzed a non-invasive test using urinary microRNAs to diagnose pancreatic cancer from different cancer stages. A support vector classifier was used to classify malignant from non-cancerous cases and it had a 97.2% and 96.3% AUC score in the training and test sets, respectively. In their study, Patel & Mukherjee^66^ developed a neural network model using four urine biomarkers (REG1A, REG1B, LYVE1, and TFF1) to predict locally progressed pancreatic cancer. The predictive ability of their urine biomarker model resulted in a 78% AUC score in pancreatic cancer diagnosis. Karar et al.^67^ developed an automated classification model using 1D-Convolutional Neural Networks and long short-term memory (LSTM) to evaluate proteomic urinary markers which achieved a high performance with an AUC of 98%. The reported studies, though they performed well, their results are lower than that observed in the current study, highlighting the necessity and benefits associated with hybrid ML approaches in non-invasive pancreatic cancer diagnosis. In addition, for medical professionals to apply this approach during patient examinations and treatments, the classification models must possess a high level of reliability, meaning they should be highly precise and accurate. Furthermore, the models ought to supply users with transparent information derived from the observed model outcomes. Nevertheless, several ML models now lack a clear explanation, which brings a challenge that ML systems are hardly implemented in medical applications. To address the fundamental limitations of existing ML models, this study used an approach that provided an understanding of the classification using SHAP. By classifying the target as either cancerous or non-cancerous, the method we used rendered progress toward achieving explainable AI for pancreatic cancer.

The implementation of SHAP in ML-based pancreatic cancer detection provides essential insights that can enhance medical decision processes. SHAP analysis can assist healthcare experts in determining key features that influence classifier response through the evaluation of clinical features which provides benefits specifically during critical complex medical cases that require advanced diagnostic solutions. For instance, in this study, SHAP analysis revealed LYVE1 and TFF1 as the most influential factors in predicting pancreatic cancer, therefore, clinicians can request extra confirmatory testing while validating these results against existing diagnostic parameters to prevent false diagnoses and unnecessary procedures. Through SHAP analysis medical professionals can diagnose cancer early by referring to the biomarker threshold values and assessing parameter significance in their clinical cases. The presence of shallow biomarker levels in SHAP results can alert a need for intensified monitoring as well as recommendations for both liquid biopsies and advanced imaging instead of invasive procedures. Furthermore, healthcare providers can utilize SHAP as a tool to validate AI diagnosis results through their analysis of the effects of input variables on predictions rather than typical black-box unpredictability. In addition, SHAP analysis allows healthcare providers to conduct risk assessments, disease diagnosis, survival analysis, and design individual treatment strategies^68–71^. For instance, when a model demonstrates that high LYVE1 indicates a high probability of a malignant condition, the medical team can design personalized treatment plans that use established successfully targeted drugs found suitable for patients with similar biomarker patterns. Moreover, medical experts use the interpretable AI methodology and empirical evidence to evaluate and defend model prediction results. Such decision-making processes become particularly essential within multidisciplinary boards which require collaborative decision-making among radiologists, pathologists, oncologists, and other healthcare professionals. Thus, the critical requirements of real-world clinical settings include accuracy, dependability, and interpretability which are the core benefits of XAI approaches.

Despite showing high accuracy in pancreatic cancer classification the proposed hybrid models still possess some limitations. The merging of different models with different functionalities requires high processing time and resources to boost computational complexity. Therefore, real-time deployment of such models in clinical environments might be difficult, particularly in locations with minimal computing capacity and limited resources. Furthermore, the models require diverse, big, and high-quality properly annotated pancreatic cancer datasets as a vital requirement to operate optimally in solving data availability problems and potential bias risks. Another major limitation is data bias that occurs due to properties within the training dataset. This research used a specified dataset which was collected from a population, with certain demographic groups and predetermined clinical characteristics. The model is likely to display limited applicability for groups of people with diverse genetic makeups, in different environmental circumstances, and at various levels of healthcare access. Furthermore, the method of collecting data might negatively affect model performance and produce inaccurate predictions when applied in larger clinical facilities, as it may raise issues in the handling of the dataset that is likely to involve missing values, mostly due to how medical facilities frequently experience data gaps in their clinical operations as a result of inaccurate clinical outcomes, or patients failing to follow protocols, or even when health facilities having logistic and operational problems. Advanced imputation methods with the potential to fill such gaps without affecting the structure of missing data points are critical as the accuracy of clinical process predictions requires robust sensitivity analysis for estimating how missing data affects predictive model performance. Therefore, future research should focus on collecting data across multiple healthcare centers representing different geographical areas and population ethnicities, and also apply more sophisticated data handling techniques to enhance the robustness and generalization capabilities of the applied models. Techniques such as the Synthetic minority oversampling technique (SMOTE) and class-weighted modeling methods could solve both bias problems as well as equalize classification outcomes.

ML-driven oncology diagnostics development requires extending the application of the ML models to diverse cancer varieties. Such models achieve generalization when their results are successful during separate validation tests involving different cancer subtypes. Therefore, transfer learning can be applied in future studies as it offers benefits in such cases due to its adaptation of single-cancer dataset-trained models through smaller datasets that can be fine-tuned using data from analogous cancers. Additionally, integrating multiple dimensional data types through multi-omics analysis can increase both the accuracy and precision of cancer subtype detection from urine biomarkers. Moreover, future studies should focus on gathering a dataset of different data forms like images, such as CT scans, PET, MRI, and histopathological imaging datasets, and developing multimodal algorithms to enhance the accuracy of predicting pancreatic cancer. Given the rising worldwide incidence of pancreatic cancer, the advancement of remote monitoring as well as early detection methods is essential for continual monitoring using Artificial Intelligence of Things (AIoT) and Internet of Medical Things (IoMT) approaches. On the other hand, providing privacy and security of data is essential for promoting confidence and greater use among healthcare professionals and patients, resulting in enhanced results and medical services.

Urine biomarkers function as predictive characteristics for early cancer detection yet they present both advantages and disadvantages in such applications. Urinary biomarkers show promise for population screening because they enable easy, financially efficient urinary analysis rather than traditional blood testing or tissue examination, however, they demonstrate limited steadiness and reproducibility associated with food consumption, metabolic factors, and liquid intake variations which can modify urine chemical content. Furthermore, uniformity in clinical applications needs standardized procedures in sample collection and processing as well as biomarker measurement methods. Our research demonstrates that urine biomarkers are effective in early cancer diagnosis yet real-world clinical validation experiments are needed to strengthen the findings of the models applied in our study and their compatibility with existing oncologic diagnosis frameworks. Managing these constraints demands collaborative actions among researchers, physicians, and AI specialists, diligent study design, comprehensive testing, and continuous improvement of ML models to boost the accuracy and validity of diagnosis for different medical conditions.

## Conclusion

This paper applied ML algorithms in the classification of pancreatic cancer using urine test results. The findings indicate that the employed approach can accurately categorize pancreatic cancer cases. This was achieved by training six ML models; LR, KNN, RF, DT, NB, and SVM as well as developing an ensemble voting classifier which was later hybridized with the six single models making them quick to learn and implement with high accuracy and precision. These classifiers achieved good performance with the results showing that the ensemble voting classifier when hybridized with random forest achieved an AUC of 99% outperforming other models. With the help of SHAP; an XAI technique, among the considered features in the whole dataset; benign sample diagnosis, TFF1, and LYVE1 were found to have a higher impact on the performance of the ML model than the rest input factors. In the future, it would be intriguing to explore the possibility of applying this framework to classify different types of diseases. This could potentially provide a cost-effective and time-saving solution in disease diagnosis increasing the survival rate and lowering the mortality rates. Therefore, a successful performance of ML models in the prediction of pancreatic cancer considering the biomarkers and other clinical parameters that contribute to diagnosis can be valuable in guiding cancer treatment in clinical practice.

## Data Availability

Data is provided within the manuscript.
